# Mass Release of *Trichogramma evanescens* and *T. cacoeciae* Can Reduce Damage by the Apple Codling Moth *Cydia pomonella* in Organic Orchards under Pheromone Disruption

**DOI:** 10.3390/insects8020041

**Published:** 2017-04-04

**Authors:** Lene Sigsgaard, Annette Herz, Maren Korsgaard, Bernd Wührer

**Affiliations:** 1 Department of Plant and Environmental Sciences, University of Copenhagen, Thorvaldsensvej 40, DK-1871 Frederiksberg C, Denmark; les@plen.ku.dk; 2 Julius Kühn Institut, Heinrichstraße 243, Annette, D-64287 Darmstadt, Germany; Annette.Herz@julius-kuehn.de; 3 Ecoadvice, Gefion, Fulbyvej 15, DK-4180 Sorø, Denmark; mak@ecoadvice.dk; 4 AMW Nützlinge GmbH, Ausserhalb 54, D-64319 Pfungstadt, Germany

**Keywords:** *Trichogramma evanescens*, *Trichogramma cacoeciae*, *Cydia pomonella*, biological control, organic production

## Abstract

*Cydia pomonella* is a major pest in apples in Denmark. *Trichogramma* spp. are known biocontrol agents of *C. pomonella* eggs and two naturally occurring species in Denmark, which are also both commercially available, were chosen for mass-release trials. *Trichogramma evanescens*, *T. cacoeciae* or a mix of the two species were evaluated for mass-release to control *C. pomonella* in two commercial organic apple orchards, one in 2012 and one in 2013, using a complete randomized block design. Pheromone disruption was used in both orchards, making the study one of the first to evaluate *Trichogramma* release under a mating disruption regime. *Trichogramma* activity was assessed using bait cards with *Sitotroga cerealella* eggs. The percent *C. pomonella* damaged fruit was recorded and the fruit yield was estimated. In 2012 cool and wet weather conditions resulted in low *Trichogramma* activity (<16% bait cards parasitized) and only *T. evanescens* was recovered from bait cards. The conditions in 2013 were warmer but *T. evanescens* was still >10 times more frequently found in bait cards than *T. cacoeciae*. There was a significant effect of the treatment and year (*p* = 0.009) and of the sampling period (*p* = 0.0008) on *Trichogramma* activity (proportion bait cards parasitized), with no significant difference between treatments in 2012. In 2013 the highest activity was found in *T. evanescens* and mixed treatments, in July reaching 69% and 47% bait cards parasitized, respectively. Fruit damage was highest in the control plots (7.1%) compared with *Trichogramma* treatments (*T. evanescens* 2.8%, *T. cacoeciae* 3.8%, mixed 3.3%) (*p* = 0.028). Yield did not differ significantly between treatments. In conclusion, *Trichogramma* mass release is a promising biocontrol method for use in the Danish climate, but further studies are needed regarding the performance of the two *Trichogramma* species (and potential other *Trichogramma* species) towards *C. pomonella* eggs in the field to identify the best biocontrol candidate.

## 1. Introduction

Denmark has 22.3% of its apple under organic production [1], and a steadily increasing demand for organic foods, with a demand for fruit alone increasing by 19% from 2013–2014 [2]. Pests and diseases cause quality and yield reductions in organic apples, with yields of grade A apples of about a third of conventional producers. In order to obtain stable or higher yields, control of pests is essential. The codling moth *Cydia pomonella* (L.) (Lepidoptera: Tortricidae) is a primary pest of apple, and in organic production, the options for its control are cultural control, conservation biological control and, since 2011, mating disruption and *C. pomonella* granulosis virus (CpGV) have been permitted. Since 2011, mating disruption has been used in most organic orchards, while few organic growers use CpGV. [3]. *Trichogramma* spp. parasitoids are important biological control agents worldwide and in Europe [4,5]. In orchards, *T. dendrolimi* Matsumura and *T. cacoeciae* (Marchal) are used to control *C. pomonella* [6,7]. Other *Trichogramma* species used in controlling this pest are *T. platneri* Nagarkatti [8], *T. minutum* Riley, and *T. pretiosum* Riley [7,9,10,11] in the United States. Recently *T. evanescens* Westwood, which occurs naturally in orchards, has become available for *C. pomonella* control in a mixture with *T. cacoeciae* [12].

*Trichogramma evanescens* and *T. cacoeciae* were selected for mass-release trials. Both species occur naturally in Denmark and are known to frequently parasitize the codling moth *C. pomonella* [13]. *T. evanescens* is relatively cold-hardy [14,15] and has higher fecundity than *T. cacoeciae* [13,15]. A mix of *T. cacoeciae* and *T. dendrolimi* is used with good results in small privately owned orchards in Germany, with only 5 ha commercial orchards officially treated [16]. Currently, the price of *Trichogramma* spp. is considered too high for use in commercial conventional orchards and *Trichogramma*’s sensitivity to sulfur, regularly used in organic orchards, is a concern. Due to a lack of alternatives, Danish organic growers are motivated to get more options to control *C. pomonella*. The present study was done to assess the potential of egg parasitoids of the genus *Trichogramma* for the control of *C. pomonella*, when applied in organic orchards using mating disruption. We hypothesized that mass release of *Trichogramma* would be able to reduce the proportion of apples with *C. pomonella* damage, and that the two selected *Trichogramma* species would both be able to perform under Danish weather conditions, possibly with the better performance of one of the species or of the species mixture.

## 2. Materials and Methods

### 2.1. Orchards

Experiments were conducted in two commercial organic orchards, one in 2012 in Høng, and one in 2013 in Kagerup, Regstrup, Holbæk. Both orchards are situated in the Central-Western part of the island of Zealand, Denmark (Høng: 55.363° N, 11.3037° E, Kagerup: 55.633° N, 11.566° E). Trees in both orchards were planted in 2006. In Høng the trees in all four blocks (experimental design described in Section 2.3) were the variety ‘Holsteiner Cox’. In Kagerup, the varieties were ‘Holsteiner Cox’, ‘Alkmene’ and two blocks of ‘Red Aroma’. Orchards were selected based on codling moth infestation in previous years and appropriate dimensions for a block trial. In 2011, both orchards had about 10% damaged apples due to *C. pomonella,* and the orchard in Høng was selected for the study in 2012. In 2012 infestations were low, but some infestation was observed in Kagerup, and this orchard was selected for study in 2013. Thermo loggers were used to record temperature in the orchards, and climate data were obtained from the University of Copenhagen (UCPH) climate station on its experimental farm in Taastrup (55.668° N, 12.305° E). In Denmark, there is only one annual generation of *C. pomonella*. Both orchards in both years of study used pheromone mating disruption of *C. pomonella* (2012: Exosec CM^®^; 2013: Isomate CLR^®^).

Experiments were initiated with the onset of *C. pomonella* oviposition according to pheromone trap captures of the first males and by an early warning from an interactive Decision Support System for pest and disease management in fruit RIMpro [17].

### 2.2. Trichogramma Spp. and Bait Cards

The *Trichogramma* were received as pupae in *Sitotroga cerealella* Olivier (Lepidoptera: Gelechiidae) eggs on folded Trichocards (TrichoKarte^®^, AMW Nützlinge GmbH, Pfungstadt, Germany). A Trichocard held ca. 3000 eggs of either *T. evanescens*, *T. cacoeciae* or a 1:1 mixture of the two species and cards were produced to have different parasitoid developmental stages (time since parasitism), which guaranteed continuous hatching of adult parasitoids for more than two weeks. The *Trichogramma* species were collected in Germany and mass cultures at AMW are frequently renewed. Trichocards were folded and stapled on both sides thus allowing emerging *Trichogramma* to pass but excluding larger insects or spiders from entering and possibly damaging the *Trichogramma* pupae. *Trichogramma* wasps were obtained from AMW Nützlinge GmbH, Pfungstadt where they had been reared in an incubator at 25 ± 1 °C, 70% ± 10% RH and a photoperiod of 16:8 (L:D). The wasps were fed on a diet of honey–gelatin. It has been noted that quality of the parasitoid may be compromised after rearing *Trichogramma* for many generations on an atypical host. Approaches taken to counter this effect include periodically switching the parasitoids to a different host [6] and populations are transferred to *C. pomonella* eggs for one generation once a year [18].

Bait cards were used to monitor *Trichogramma* activity and were baited with ca. 3000 three-day-old *S. cerealella* eggs. Bait cards were also folded cardboard cards. To protect *S. cerealella* eggs from predatory insects, bait cards were also stapled on the sides still allowing the *Trichogramma* to enter. Until field use, bait cards were stored at 8 °C and 60% relative humidity to prevent *S. cerealella* eggs from hatching.

The two *Trichogramma* species were distinguished by their colour, and the morphology of the antenna of male *T. evanescens* [19]. *T. cacoeciae* is a thelytokous species where no males are present.

Results of mass release of *Trichogramma* may be affected by naturally occurring *Trichogramma*. Both in 2012 and 2013 bait for *Trichogramma* was also done in a part of the two orchards without *Trichogramma* mass release (at least 100 m away), and in three other orchards on Zealand (Ventegodtgaard, Strandegaard and Kyse) in spring (late May/early June), early summer (late June) and late summer (late July/early August). In 2012 baiting was done with *S. cereallela* eggs and in 2013 baiting was done with *C. pomonella* eggs. Since baitings for naturally occurring *Trichogramma* were unsuccessful, it can be assumed that the natural population was low and would not affect the experiment [20].

### 2.3. Experimental Design and Field Assessment

A randomized complete block (RCB) design with four blocks was used both years. There were four treatments *T. evanescens*, *T. cacoeciae*, a 1:1 mixture of the two and a no release control. Each treatment was replicated once per block, the block being a tree-row of one apple variety and blocks were separated by at least one tree-row. Treatment plots within each block were 20 m long equivalent to 18 trees and with at least five trees between treatments. In each treatment plot, except control plots, a Trichocard was placed on every third tree (6 Trichocards per treatment plot). They were placed 1.5–2 m high on a branch 30–80 cm from the trunk. Trichocards were fixed to the branch by green twine. In 2012 Trichocards were set out three times with ca. three weeks interval on 4 June, 24 June and 17 July. In 2013 Trichocards were only set out twice, on 17 June and 5 July. Eight Trichocards for each treatment were checked for hatch and *Trichogramma* species composition in the laboratory.

In each treatment plot, a bait card with *S. cereallela* eggs was placed on each of eight different trees, avoiding trees with a Trichocard, to record *Trichogramma* parasitism. Bait cards were labeled by treatment and date and in both years cards were set out on the same day as the Trichocards and collected after 72 h. In 2012 an additional baiting was done 24–27 July where only four bait cards were used per treatment plot. This baiting was done as hardly any parasitism was observed on 20 July 2012, most likely due to rainy and windy weather conditions, and for later statistical analysis data from 20 July 2012 was excluded, leaving three sampling periods for analysis.

Collected bait cards were checked for presence of any arthropods, which were recorded and removed before transport to the lab in a cooling bag with cooling elements maintaining ca. 12 °C. Bait cards were placed individually in Petri-dishes sealed with parafilm, labeled with block and treatment and kept at 20 °C, L:D 16:8 for at least two weeks regularly checking for *Trichogramma* emergence. In a few cases all eggs on a bait card had been eaten, typically if an earwig had entered the bait card. These cards were recorded as missing.

The damage from *C. pomonella* was identified by entry or exit holes on apples and when needed, such as in cases where holes were by the calyx or stem end of the fruit and hard to detect, the damage was confirmed by dissecting apples. Early in the season holes caused by apple sawfly *Hoplocampa testudinea* (Klug) may be mistaken for *C. pomonella* damage, but the damage caused by this pest looks different and apples affected by apple sawfly normally fall off the tree by the so-called “June drop”, before our damage assessment. On 19 July 2012 *C. pomonella* infestation was assessed in all blocks. In each treatment plot, 10 apples from 10 randomly selected trees were visually assessed for *C. pomonella* damage, (400 fruits per treatment for the full four-block experiment). Total yield was assessed on 21 August 2012 from counts of the number of all apples per branch on two randomly selected branches per plot. In 2013, fruit damage was assessed 1 July 2013 and yield was assessed 29 August 2013. This was done as in 2012, but in 2013 damage was assessed on 10 apples from eight randomly selected trees per treatment plot (320 fruits per treatment for the full four-block experiment), and total yield was assessed based on counts of all fruits on eight to 10 branches per treatment plot. In 2013 yield per tree was estimated by multiplying yield per branch by the average number of branches per tree. Mid-season damage assessment before growers start thinning damaged fruit gives a good assessment of the pest pressure. Late damage assessment is underestimating damage as a result of organic growers’ careful thinning of damaged fruits. Yield assessment before harvest, on the other hand, gives a better assessment of final harvest.

### 2.4. Data Analysis

The proportion of bait cards with *Trichogramma* parasitism (irrespective of the number of eggs parasitized) was analysed as it reflects the wasps’ ability to disperse, find and parasitize hosts. Proportions were analyzed assuming a binomial distribution using SAS Proc GLIMMIX [21]. The full model included sampling period (week (year)), year, treatment and their interactions, with block as a random factor. The proportion of *T. cacoeciae* of total wasps emerged was used to analyze the relative success of the two species. This was tested in 2013, as in that year both species emerged. Proportions of apples damaged by *C. pomonella* were analyzed assuming a binomial distribution using SAS Proc GLIMMIX. In the model, the logit of the binomial probability depended on the fixed effects of sampling period week, treatment, and the two-way interaction, with block as a random factor. A Chi-square test was done to compare the proportion of bait cards found by the two species of *Trichogramma* by treatment and week. Total yield (apples per branch) was square-root transformed prior to analysis and analyzed as a function of treatment, year and the interaction between treatment and year, with block as a random factor using SAS Proc Mixed [21]. Non-significant interaction effects of fixed effects were removed from the final models.

## 3. Results

### 3.1. Trichogramma Releases

The summer of 2012 was cold and wet (average temperatures (±SD) and precipitation from the UCPH climate station; June: 14.1 ± 3.3 °C and 74 mm, July: 17.6 ± 3.5 °C and 60 mm, August 18.2 ± 3.7 °C and 32 mm), which resulted in low *C. pomonella* infestation. *Trichogramma* activity was also low, as determined from parasitism found in bait cards, with only 136 *Trichogramma* emerging from bait cards across all the four dates of sampling. Only *T. evanescens* emerged from the bait cards in 2012. The summer of 2013 was warmer and drier (average temperatures (±SD) and precipitation from the UCPH climate station; June: 16.3 ± 3.2 °C and 48 mm, July: 19.3 ± 3.7 °C and 17 mm, August 18.4 ± 3.4 °C and 34 mm), and *C. pomonella* infestation was higher, and from the first collection date 20 June, a total of 810 *T. evanescens* and six *T. cacoeciae* emerged from all bait cards. *T. evanescens* continued to be the dominant species found on bait cards (8 July: 1250 *T. evanescens* and 114 *T. cacoeciae* emerged).

**Figure 1:**
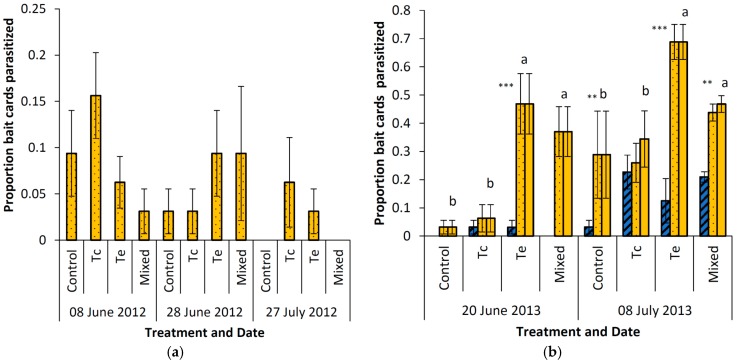
*Trichogramma* activity assessed as mean proportion (±SE) of bait cards being parasitized with *Trichogramma evanescens* (orange and dotted), *T. cacoeciae* (blue and hatched) and the total proportion bait cards being parasitized (purple) in a completely randomized block experiment with three treatments; mass release using Trichocards^®^ of *T. evanescens* (Te), *T. cacoeciae* (Tc), a mix of the two species (Mixed) and a control in (**a**) 2012, where only *T. evanescens* emerged and no significant differences were found between treatments; (**b**) in 2013 where both species emerged. Letters indicate significant differences between total proportion bait cards parasitized based on pairwise comparisons of ls-means. Asterisks indicate significant differences (Chi-square test, * *p* <0.05, ** *p* <0.01, *** *p* <0.001) between proportion cards found by the two species within a treatment.

There was a significant interaction effect of the treatment and year (*F*_3,66_ = 4.23, *p* = 0.009) and a significant main effect of the sampling period (week(year)) (*F*_2,66_ = 6.28, *p* = 0.0008) on the proportion of parasitized bait cards (Figure 1). Pairwise comparisons of ls-means showed no significant differences between treatments in 2012, while in 2013 the highest parasitism was found in *T. evanescens* and mixed treatments, with *T. evanescens* higher than the mixed treatment, but not statistically significantly (*t* = 1.87, *p* = 0.066). The *T. cacoeciae* treatment was not significantly different from the control. In 2012, the proportion of bait cards parasitized was significantly higher in the first sampling period (7.6%) than the last sampling period (2.0%) (*t* = 2.07, *p* = 0.042), while the intermediate period was not different from the other two periods (5.5%). In 2013 the proportion of cards parasitized in the first period was less than half (19.2%) of the second sampling period (43.4%) (*t* = −3.81, *p* = 0.0003). In 2013 both *T. evanescens* and *T. cacoeciae* emerged from bait cards, and there was a significant effect of the treatment and period on the proportion of bait cards found by *T. cacoeciae* with a higher proportion emerging in the *T. cacoeciae* treatment and more in July, but no interaction effect of the treatment and period. From the first to second sampling period in 2013, the proportion of cards found by *T. cacoeciae* increased from 0.64% to 6.62% (*F*_16,28.2_ = 28.8, *p* < 0.0001). The proportion of cards found by *T. cacoeciae* was highest in the *T. cacoeciae* treatment (18.4%), intermediate in the mixed treatment (4.7%) and low in the *T. evanescens* (0.2%) and control (0.1%) treatments. The proportion of bait cards found by *T. evanescens* was significantly higher in all treatments except the *T. cacoeciae* treatment (Figure 1).

A laboratory check in 2013 of eight Trichocards, with *T. cacoeciae*, *T. evanescens* and a mix of the two species, showed that *T. cacoeciae* cards produced an average of 1737.5 ± 69.5 parasitoids of which 99.7% ± 4.0% were *T. cacoeciae*. For *T. evanescens* cards, 1475.0 ± 55.9 wasps hatched of which 100% ± 3.8% were *T. evanescens*. From the mixed Trichocards, 1975 ± 50.8 parasitoids hatched of which 54.6% ± 2.6% were *T. evanescens* (males and females) and 45.4% ± 2.6% *T. cacoeciae* (only females). Plots were as large as the orchard size permitted (20 m long and separated from other treatments, see details in Section 2.3), but in 2013, after the second augmentative release, the proportion of the bait cards found by *T. evanescens* was 26% and 29% in the *T. cacoeciae* and control treatments, respectively, indicating the dispersal of *T. evanescens* to these plots.

### 3.2. Fruit Damage and Yield

**Figure 2:**
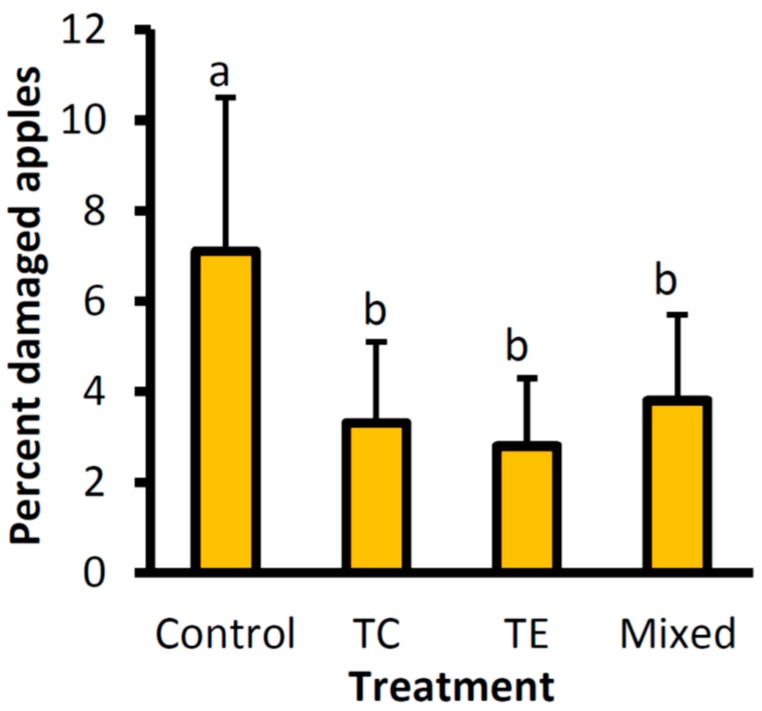
Average percent apples damaged by *C. pomonella* (±SE) in a completely randomized block experiment with four treatments; mass release using Trichocards^®^ of *T. evanescens* (Te), *T. cacoeciae* (Tc), a mix of the two species (Mixed) and a control for years 2012 and 2013.

The effect of treatment on fruit damage was significant (*F*_3,27_ = 3.55, *p* = 0.028), with a higher percent of damaged apples in the control (7.1% ± 3.4%) than in *Trichogramma* treatments (*T. evanescens* (2.8% ± 1.5%) (*t* = −2.73, df = 27, *p* = 0.01)*,* mixed (3.8% ± 1.9%) (*t* = −2.12, df = 27, *p* = 0.04), and *T. cacoeciae* (3.3% ± 1.8%) (*t* = 2.32, df = 27, *p* = 0.03)) (Figure 2). Fruit damage was significantly higher in 2013 (5.7% ± 2.7%) than in 2012 (2.8% ± 1.4%) (*F*_1,27_ = 7.66, *p* = 0.010), but there was no interaction effect between the damage and year.

In both years the total yield was estimated as apples per branch. In 2012 the total yield was: apples/branch: *T. evanescens* 1.6 ± 0.6 apples, *T. cacoeciae* 2.4 ± 0.5 apples, mixed 1.8 ± 0.8 apples and control 1.6 ± 0.4 apples. The total yield by late August 2013 was also estimated for whole trees. The estimated yield per branch (and per tree) in 2013 was: *T. evanescens* 3.62 ± 1.33 (76.45 ± 42.10) apples, *T. cacoeciae* 3.89 ± 1.30 (53.99 ± 24.55) apples, mixed 2.78 ± 0.72 (77.98 ± 44.50) apples and control 3.12 ± 0.78 (48.17 ± 22.95) apples. There was no significant difference between treatments (*F*_3,24_ = 0.19, *p* = 0.90) or years (*F*_1,24_ = 0.04, *p* = 0.83) in terms of the total yield.

## 4. Discussion

In both the 2012 and 2013 treatments, the *T. evanescens* and mixture of *T. evanescens* and *T*. *cacoeciae* showed higher parasitoid activity as measured by the proportion of bait cards parasitized. *T. evanescens* was dominant among emerging wasps, and the *Trichogramma* species mixture treatment did not improve parasitism on bait cards. The difference in the performance of the two *Trichogramma* species may suggest that *T. evansescens* is better adapted to the Danish climate, as *T. cacoeciae* performs well against *C. pomonella* in Germany, where the summer temperatures are higher (average temperatures in Frankfurt, Germany, ca. 800 km south of our field sites: June 17.3 **°**C, July 19.5 °C, August 19.1 °C). In 2013 the proportion of *T. cacoeciae* found in bait cards increased as the season progressed and temperatures increased. Regional differences in *Trichogramma* performance are also known from the US, where mass release of the native *T. platneri* outperformed three other *Trichogramma* species [11], even the arboreal species *Trichogramma minutum*, which is indigenous to the eastern US (3500 km distance). The author suggests its performance may have been compromised by the much drier climate of California [11]. It is, however, also possible that bait cards with *Sitotroga* eggs were more attractive to *T. evanescens* than to *T. cacoeciae* and therefore *T. evanescens* might prevail in this experimental design. However, no other method for measuring parasitoid activity was feasible because codling moth density was very low and egg parasitism could not be assessed directly. 

All treatments with *Trichogramma* had significantly lower damage than the control, though observed damage was low. Growers in Danish organic orchards are very keen on sanitizing the orchard by hand, removing any fruit with signs of disease or insect attack such as *C. pomonella*, as alternatives for control are few, so results provided a conservative estimate of *C. pomonella* infestation in the orchards. Both orchards in both years of study used mating disruption of *C. pomonella* since this became allowed in 2011, which most likely reduced *C. pomonella* infestation.

Damage reduction is equivalent to the 50%–70% reported from other studies [4,18], and the damage level of 1%–3% is equivalent to that reported from other studies. For conventional apples <1% damaged by *C. pomonella* is accepted, but the result of *Trichogramma* release still shows that a large improvement over the current situation can be obtained in organic orchards. Damage assessment is difficult as it will be influenced by growers’ hand thinning of infested fruits. A late assessment of damage provided very low damage and is not included here. Thus our comparisons of damage between treatments are based on assessments made in July, before most hand thinning of the fruit was done. For future studies, an assessment of the proportion of damaged fruit removed during hand thinning should be done, and if possible, including more than one orchard per year would also improve, to some extent, the scientific accuracy.

A difference in the total yield assessed could occur as a result of infested fruit dropping from the trees, and other infested fruit being removed by hand thinning. However, the total yield was not significantly different between treatments, and variation was very large. Factors other than *C. pomonella* such as any other pests or diseases as well as orchard management would have affected yield, contributing to the variability of the results.

The results show that *Trichogramma* mass release against *C. pomonella* can be successfully used in orchards where mating disruption is also used.

## 5. Conclusions

In conclusion, *Trichogramma* mass release is a promising strategy for use in the Danish climate. In cool years, such as 2012, augmentative releases of *Trichogramma* will be unnecessary as *C. pomonella* infestation remains low, while in warmers years, such as in 2013, results obtained with *Trichogramma* treatments are equivalent to results from studies of other *Trichogramma* species elsewhere [4,18,22,23]. Results show that *Trichogramma* mass release can be successfully used in orchards in which the mating disruption of *C. pomonella* is also used. Release may be guided by the orchards’ history of infestation with *C. pomonella*, and timing of releases can be decided based on the pests’ phenology. Though *T. evanescens* was more active on bait cards, damage assessment showed no significant difference between the three *Trichogramma* treatments. However, it may be possible to identify *Trichogramma* species or strains better adapted to cool climates in Northern Europe. Though the price of *Trichogramma* is high compared to insecticide, it provides an important alternative for organic pome fruit growers, and with increasing environmental awareness and stricter regulations, it becomes increasingly important for plant producers that biological control methods are available and documented in field trials.
